# Human Pharmacokinetic Profiling and Comparative Analysis of Mangiferin and Its Monosodium Derivative from *Mangifera indica* Extracts Using UHPLC-MS/MS with ^1^H NMR and MALDI-TOF Confirmation

**DOI:** 10.3390/molecules30030461

**Published:** 2025-01-21

**Authors:** David Fuentes-Rios, Alvaro Sanchez-Rodriguez, Laura Lopez-Rios, Eduardo Garcia-Gonzalez, Miriam Martinez-Canton, Victor Galvan-Alvarez, Angel Gallego-Selles, Marcos Martin-Rincon, Jose A. L. Calbet, Tanausu Vega-Morales

**Affiliations:** 1 Nektium Pharma SL, C/Las Mimosas 8, Polígono Industrial Arinaga, 35118 Las Palmas, Spain; dfuentes@nektium.com (D.F.-R.); asanchez@nektium.com (A.S.-R.); llopez@nektium.com (L.L.-R.); 2 Department of Organic Chemistry, Faculty of Sciences, University of Malaga, Campus de Teatinos s/n, 29071 Málaga, Spain; 3 Department of Physical Education and Research Institute of Biomedical and Health Sciences (IUIBS), University of Las Palmas de Gran Canaria, Campus Universitario de Tafira s/n, 35017 Las Palmas de Gran Canaria, Spain; eduardo.garcia124@alu.ulpgc.es (E.G.-G.); miriammartinezcanton@gmail.com (M.M.-C.); victor_galvan@hotmail.es (V.G.-A.); angelgallegoselles@hotmail.com (A.G.-S.); marcos.martinrincon@gmail.com (M.M.-R.); lopezcalbet@gmail.com (J.A.L.C.); 4 Instituto Universitario de Estudios Ambientales y Recursos Naturales (i-UNAT), University of Las Palmas de Gran Canaria, Campus Universitario de Tafira s/n, 35017 Las Palmas de Gran Canaria, Spain

**Keywords:** mangiferin, salification, bioavailability, pharmacokinetic, liquid chromatography, mass spectrometry

## Abstract

Mangiferin, a glucosyl xanthone, is a plant metabolite with promising nootropic and ergogenic properties. However, its poor and inconsistent systemic bioavailability limits its therapeutic potential. Additionally, the pharmacokinetics of mangiferin from mango leaf extract formulations remain uncharacterized in humans. This study validated a UHPLC-MS/MS method and conducted a human pharmacokinetic study approved by an ethics committee. The bioavailability of mangiferin and its monosodium salt was assessed from two standardized mango leaf extracts: MLE60, standardized to 60% mangiferin but practically insoluble in water, and MLES, the water-soluble monosodium salt form, also standardized to 60%. Twelve participants (six females) received oral doses of each extract in a crossover design with a 7-day washout period. Plasma analysis showed significantly higher AUC and C_max_ values with MLES than MLE60, while T_max_ and T_1/2_ were similar. MLES demonstrated a 2.44-fold increase in AUC_0–24h_ compared to MLE60 (*p* = 0.0029 **), indicating improved bioavailability. This study highlights the salification method as a simple strategy to enhance mangiferin bioavailability, enabling broader applications in beverages and other products where solubility is a limitation.

## 1. Introduction

The species *Mangifera indica* L. of the Anacardiacea family, popularly known as mango, is native to tropical Asia and is currently cultivated worldwide to produce flavorful mango fruit [1,2]. Scientific interest in the medicinal properties of the leaves, bark, and fruit of this plant has recently emerged. Ethnobotanical research has revealed that mango leaves play a significant role in traditional medicine, especially in Southeast Asia and Africa [3]. They are commonly used to treat a variety of conditions, including diabetes mellitus, burns, inflammation, diarrhea, dysentery, leukocytosis, and several other ailments [4].

Phytochemical studies of mango leaves reveal the presence of several bioactive phenolic compounds, including phenolic acids, flavonoids, benzophenones, gallotannins, and xanthones [5,6,7,8]. The glucosyl-xanthone mangiferin, one of the most abundant and bioactive phytochemicals in mango leaves (36.9–67.2 mg/g, favoring young leaves) [8,9], is a well-known polyphenolic compound that has been extensively investigated for its pleiotropic effects and remarkable pharmacological properties, which include potent antioxidant, anti-inflammatory, antidiabetic, hepatoprotective, antibacterial, antiviral, analgesic, antimalarial, anti-tumor, neuroprotective, neuromodulatory, and ergogenic activities [10,11,12,13].

Recently, a novel herbal extract from mango leaves, enriched with mangiferin, has been developed and patented in the USA by del Río et al. [14]. This herbal formulation, marketed under the name Zynamite^®^, has demonstrated phytonootropic properties, improving mental performance both in vitro and in vivo [15]. Additionally, Zynamite^®^ has been shown to enhance exercise performance when combined with other polyphenols [16,17,18]. Despite the claimed physiological activities, the pharmacokinetics of mangiferin from mango leaf extracts have not yet been characterized in humans. Furthermore, the use of pure mangiferin via the oral route has been limited due to its poor and fluctuating systemic bioavailability [19,20], which is generally attributed to its low aqueous solubility (0.111 mg/mL) [21], limited lipophilicity [22], which results in insufficient intestinal membrane permeability, and its significant P-glycoprotein (P-gp) efflux [23,24].

Various strategies have been employed to improve the bioavailability of mangiferin, including molecular inclusion in cyclodextrins [25], combinations with natural polymers such as pectins or chitosan [26], lipid emulsions containing surfactants [27], and complexation into lipo- or phytosomes [28,29]. Bioavailability may also be enhanced through positive interactions and synergies between two or more ingredients in complex botanical formulations [24,30]. Given the intricacy of herbal extracts, it is also possible that some constituents of the mango leaf may modulate the absorption of mangiferin [23]. Previous reports have indicated an increase in the bioavailability of mangiferin from raw herbal extracts or polyherbal formulations compared to that of pure mangiferin [20,24].

In line with the above, this study evaluates two different grades of mango leaf extracts in a human pharmacokinetic study. The first product (MLE60) was an aqueous extract of *Mangifera indica* leaves, purified to 60% (*w*/*w*) mangiferin through crystallization. In the second product (MLES), a salification reaction was applied to the MLE60 extract, resulting in the formation of mangiferin monosodium salts while maintaining the same 60% (*w*/*w*) concentration. As a result of this modification, MLES exhibits a water solubility of up to 10 mg/mL at room temperature, significantly higher than that of MLE60, which demonstrates solubility levels of 0.1–0.5 mg/mL at room temperature that are very close to those of pure mangiferin [21].

This increased solubility of MLES is expected to enhance the absorption of mangiferin in the gastrointestinal tract [30] and, therefore, further improve the bioavailability and efficacy of the extract. Salification reactions have been demonstrated and applied to many botanical extracts and drugs, including mangiferin [31,32]. The increased absorption of the salified product is closely associated with the higher solubility of the formed salts compared to the starting molecules.

Finally, most published methods for determining mangiferin in plasma samples rely on protein precipitation using organic solvents and centrifugation. While techniques such as protein precipitation (PPT) and liquid–liquid extraction (LLE) are effective, they have limitations, including inefficient phospholipid removal, which contributes to matrix effects and may lead to variable mass spectrometry results [33]. Additionally, both PPT and LLE are typically performed in multiple steps, which often results in the poor recovery of analytes and reduced sample throughput. In this context, Ostro™ pass-through sample preparation plates facilitate the integration of liquid–liquid extraction of mangiferin from plasma into an organic solvent, along with protein precipitation, phospholipid removal, and particulate filtration within a single sample preparation step.

The objectives of this study were twofold. First, to develop, optimize, and validate a simple yet efficient analytical method for determining mangiferin in human plasma samples, ensuring high reproducibility, sensitivity, and throughput. Second, to assess the relative bioavailability of mangiferin and its monosodium salt from two distinct mango leaf extracts through a pharmacokinetic study conducted in human subjects, in accordance with the Declaration of Helsinki, and approved by an ethical committee (CEIH-2023-05-R, 1 June 2023).

## 2. Results

### 2.1. Sample Preparation and UHPLC-MS/MS Analysis

Various combinations of solvents, additives, and volumes were assessed to reconstitute the samples following the Ostro™ pass-through protocol and evaporation to dryness. A mixture of MeOH:H_2_O (1:1, *v*/*v*), containing 0.1% (*v*/*v*) formic acid and 20 mM ammonium formate, facilitated the complete redissolution of the samples while providing good chromatographic resolution for both mangiferin and taxifolin (internal standard, IS), achieving better signal-to-noise ratios than those obtained when redissolving the samples in pure methanol, acetonitrile, or combinations of either solvent with water. Different reconstitution volumes were evaluated (100, 200, 300, and 400 µL). Using 100 µL, we concentrated the samples twofold without affecting the recovery of mangiferin. Given the need for low quantification limits to determine mangiferin in plasma samples, we chose the lowest volume (100 µL) for redissolving the samples prior to UPLC-MS/MS analysis.

Mass spectrometry parameters were also carefully optimized, including source voltages, gas temperatures, flow rates, ionization modes, analyzer resolution, fragmentation transitions, and scanning modes. Both negative and positive Electrospray Ionization (ESI) modes were evaluated with mangiferin and IS perfused separately into the mass spectrometer. The best signal-to-noise ratios for both compounds were obtained in the negative ionization mode. In addition, mangiferin was slightly reluctant to ionize at low capillary voltages and desolvation temperatures; thus, relatively high values of both parameters were chosen, although this may have a negative impact on the matrix effect. On the other hand, the negative ionization mode is generally considered to be more specific and less prone to ion suppression [34], which may counteract the selection of a less selective capillary voltage in the ESI chamber.

### 2.2. Method Validation

#### 2.2.1. Specificity, Linearity and Sensitivity

**Figure 1:**
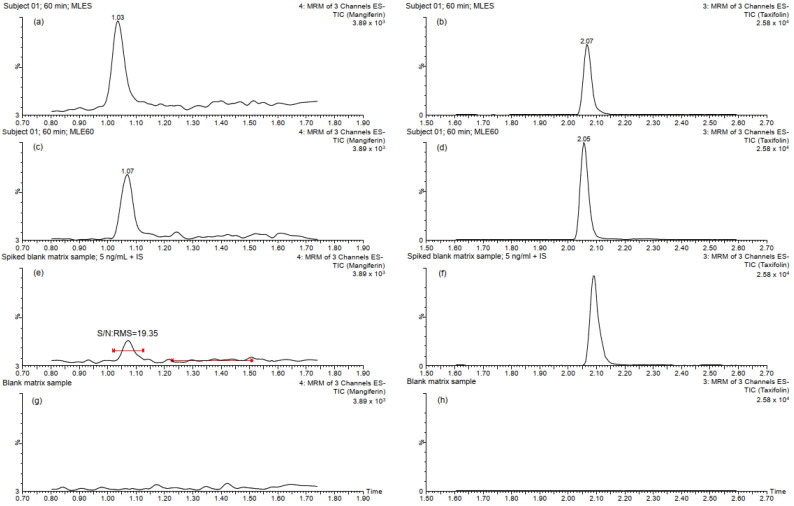
Chromatograms of plasma samples evaluated for specificity: (**a**–**d**) correspond to TIC chromatograms of mangiferin and IS obtained from the 60 min plasma samples of a subject treated with either MLES or MLE60; (**e**,**f**) correspond to TIC chromatograms of mangiferin at the LLOQ (5 ng/mL) and IS (125 ng/mL) obtained from spiked blank plasma samples, respectively; (**g**,**h**) correspond to the chromatograms obtained from the blank plasma samples used to construct the calibration curves and the QC samples.

Figure 1 shows the chromatograms of the three sample types analyzed for specificity. Notably, no interfering endogenous substances were detected at the retention times of either mangiferin or the internal standard (IS), demonstrating the high selectivity of the method. In addition, the chromatographic profiles and UV spectra obtained using a PDA detector connected in-line before the mass spectrometer confirm the effectiveness of both the sample preparation steps and the chromatographic separation. These results highlight the successful removal of potential interferences and ensure the accurate separation of mangiferin from endogenous plasma components (see Supplementary Data).

The method demonstrated good linearity over the selected concentration range (5–1000 ng/mL), with coefficients of determination (R^2^) ≥ 0.99. The lower limit of quantification (LLOQ) was 5 ng/mL, which is sufficiently low to quantify mangiferin in the human plasma samples collected in this pharmacokinetic study [19]. The intra-day precision (relative standard deviations, or RSDs) and accuracy at the LLOQ were within the acceptable criteria (8.28% RSD and 95.74% accuracy). The response of mangiferin at the LLOQ was at least 10 times greater than that of the baseline noise.

#### 2.2.2. Accuracy and Precision

Table 1 summarizes the data obtained for accuracy and precision at the selected QC concentration levels. The intra-day and inter-day precision (RSDs) were less than 11.91% and 9.47%, respectively. Accuracy data ranged from 97.93% to 103.57%. The results obtained for both parameters were within the acceptance limits, indicating that the method is reliable and reproducible.

#### 2.2.3. Matrix Effect, Recovery, and Stability

Table 2 highlights the data obtained in determining extraction recovery, matrix effect, and stability in the QC samples. As can be seen, recoveries ranged from 74.41% to 81.11%, with RSDs below 12.42%. The data obtained for this parameter suggest that mangiferin is not retained during protein precipitation or phospholipid purification, which represent probably the most critical steps considering the potential interactions between polyphenols and macromolecules [35,36].

The matrix effect analysis indicated minimal interference from the components of the plasma samples. Matrix effects ranged from 96.51% to 109.99%, with relative standard deviations (RSDs) below 11.44%. The use of Ostro™ pass-through plates, combined with optimized chromatographic and MS/MS conditions, is highly effective in minimizing ionic suppression of mangiferin in the ESI chamber.

Finally, the stability tests demonstrated that mangiferin is stable throughout the analytical process. These results align well with those reported by other researchers [20,31,37].

### 2.3. Analysis of Plasma Samples

#### 2.3.1. Pharmacokinetic Parameters

The main pharmacokinetic parameters obtained for the two treatments are summarized in Table 3. Dose-normalized AUC_0–t_ (n = 12) was analyzed at different time sampling points: AUC_0–1h_, AUC_0–2h_, AUC_0–4h_, AUC_0–6h_, AUC_0–10h,_ and the final time point, AUC_0–24h_. Other pharmacokinetic parameters, such as the maximum (or peak) mangiferin plasma concentration (C_max_), the time at which C_max_ is observed (T_max_), the half-life (T_1/2_), the apparent volume of distribution (V_z_/F), and clearance (Cl/F), were also determined.

**Figure 2:**
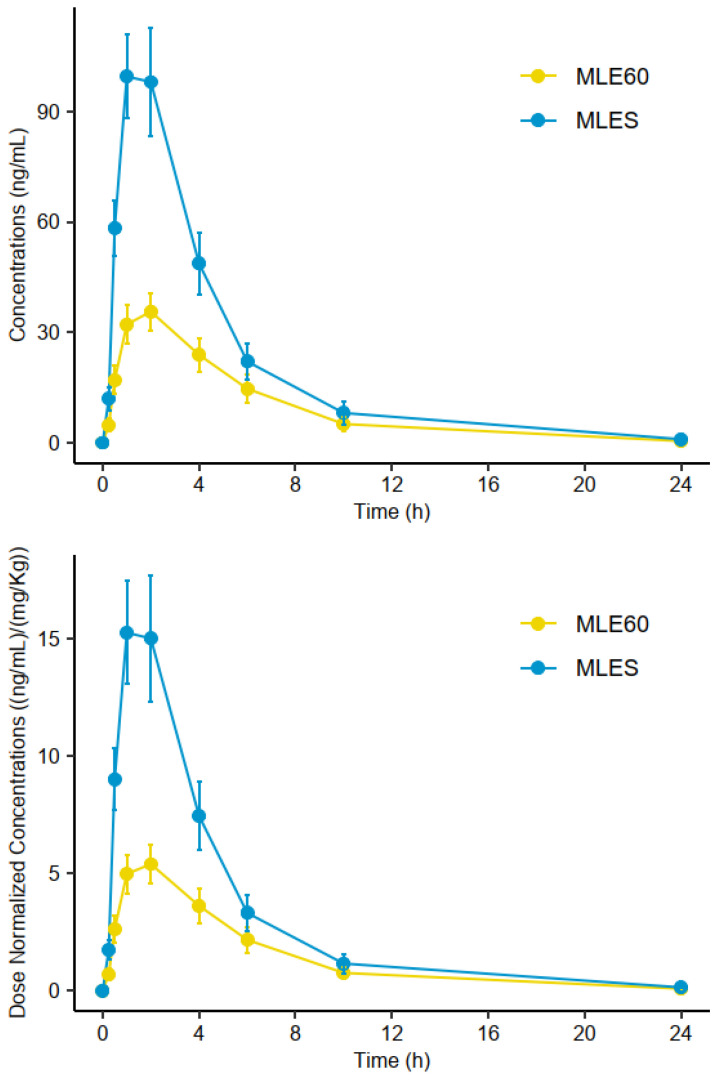
Comparisons of arithmetic mean concentration (**upper**) and dose-normalized concentration (**lower**)—time profiles of mangiferin between MLE60 (yellow) and MLES (blue) treatments. Error bars represent the standard deviation.

Analysis of the pharmacokinetic curves showed that MLES resulted in significantly higher normalized AUC_0–24h_ and C_max_ than MLE60 did (Figure 2). Since the amounts of mangiferin administered in each treatment were nearly identical (478.8 mg for MLES versus 480.4 mg for MLE60), the comparison between normalized and non-normalized curves yielded almost identical profiles. Subject-by-subject analyses of the curves revealed high inter-individual variability in the absorption of mangiferin; however, the absorption of this xanthonoid was consistently higher in each of the subjects administered MLES than in those administered MLE60. Furthermore, when the pairwise Wilcoxon–Mann–Whitney test was utilized to analyze the differences in mangiferin absorption between males and females, no significant difference was found for any of the groups (data and figures are shown in the Supplementary Data).

The T_1/2_ values obtained were 2.98 ± 2.04 h and 3.19 ± 1.68 h for MLES and MLE60, respectively. T_1/2_ is defined as the time required for the concentration of the bioactive compound in plasma to be reduced by 50%, indicating that mangiferin is metabolized similarly by both treatments. The C_max_ values for MLES and MLE60 were observed 2 h after the ingestion of each treatment; however, the narrower range observed for MLES may suggest that absorption is more acute with the soluble reference. The apparent volume of distribution (V_z_/F) for both treatments—58.0 ± 41.8 L/kg for MLES and 157.0 ± 72.9 L/kg for MLE60—indicates that mangiferin from the salified version of the extract has a more limited distribution in the body, implying that a lower dose is needed to achieve a specific plasma concentration of mangiferin. The data obtained for clearance (Cl/F) suggest that mangiferin may be eliminated from plasma more efficiently when taken as MLE60 than as MLES, given that the elimination rate is higher (40.1 ± 39.2 L/h/kg versus 16.7 ± 11.4 L/h/kg).

#### 2.3.2. Relative Bioavailability (F_rel_) Assessment

To estimate the relative bioavailability (F_rel_) or bioequivalence between MLE60 and MLES, the dose-normalized AUC_0–t_ obtained for both treatments was compared at each time point (i.e., AUC_0–1h_, AUC_0–2h_, AUC_0–4h_, AUC_0–6h_, AUC_0–10h,_ and AUC_0–24h_). The results are presented in Table 4.

**Figure 3:**
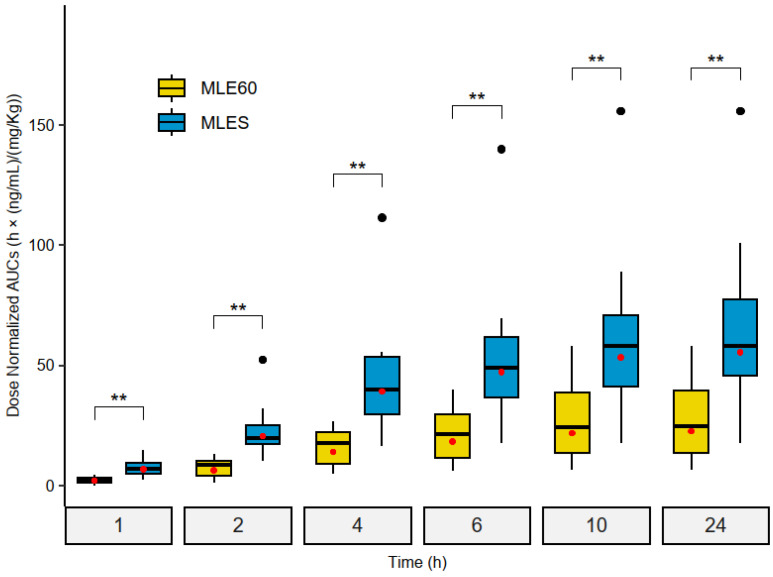
Wilcoxon–Mann–Whitney box-and-whisker plots for pairwise samples. Comparisons of dose-normalized AUC_0–t_ (t = 1, 2, 4, 6, 10, and 24 h) between MLE60 and MLES. Red dots represent geometric mean and asterisks Wilcoxon-Mann-Whitney pairwise test significance levels (** *p* < 0.01).

Consistent with the AUC_0–24h_ dose-normalized results, mangiferin from MLES is absorbed 2.44 times more than that from MLE60 (*p* = 0.0029 **). When reviewing the dose-normalized AUC at each time sampling point, the results reveal that the differences in the absorption of mangiferin are greater within the first two hours following the ingestion of the extracts. In fact, 1 h after the administration of the treatments (AUC_0–1h_), mangiferin is absorbed 3.19 times more from MLES than from MLE60, whereas 2 h after administration, the absorption is 3.24 times higher (Figure 3).

### 2.4. Characterization of Mangiferin Monosodium Salt

**Figure 4:**
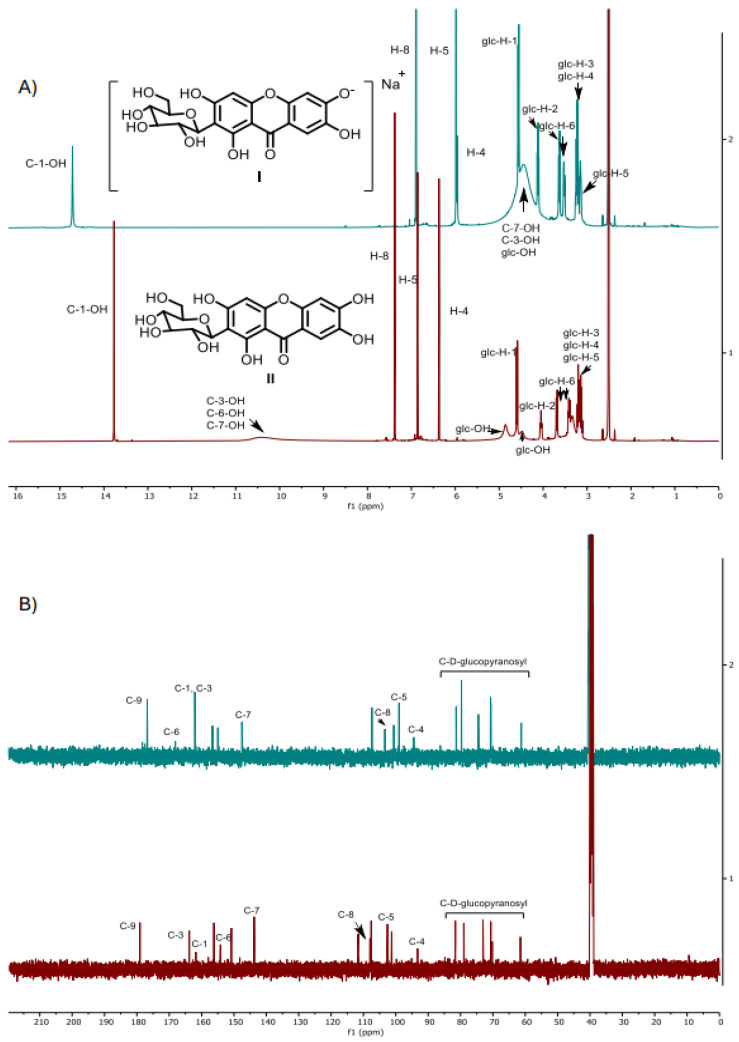
Comparative molecular structure of mangiferin monosodium salt (I, green spectra) and mangiferin (II, red spectra) by (**A**) ^1^H-NMR (DMSO-d_6_) and (**B**) ^13^C-NMR (DMSO-d_6_) analysis.

^1^H nuclear magnetic resonance (NMR) and ^13^C-NMR analysis were critical in confirming the structure of the mangiferin monosodium salt formed in the MLES extract. The ^1^H-NMR spectra recorded for both treatments are slightly different and show significant chemical shifts (Figure 4A). The proton of the hydroxyl group in position 1, present at 13.7 ppm for the mangiferin obtained from MLE60, shifts to 14.7 ppm in the mangiferin monosodium salt acquired after the salification process. Moreover, the signals related to the aromatic hydrogens [38,39] observed at 7.4, 6.8, and 6.4 ppm are now recorded at 6.9, 5.98, and 5.96 ppm, respectively. This chemical shift can also be observed in the corresponding carbons at these positions (4 and 5, positions of rings A and B, respectively) and at C-6, which now appears at a higher chemical shift (Figure 4B), as has been documented in the literature [40,41]. This phenomenon can be attributed to the localized negative charge on the 6-position alkoxy ion (R-O^−^), which increases the electron density around neighboring hydrogen atoms, thereby shifting their NMR signals to lower ppm values (the shielding effect). Finally, the broad signals observed between 10.6 and 10.0 ppm, corresponding to hydroxyl groups of aromatic rings, are now located between 5.0 and 4.0 ppm, indicating that the loss of a proton was also noted.

**Figure 5:**
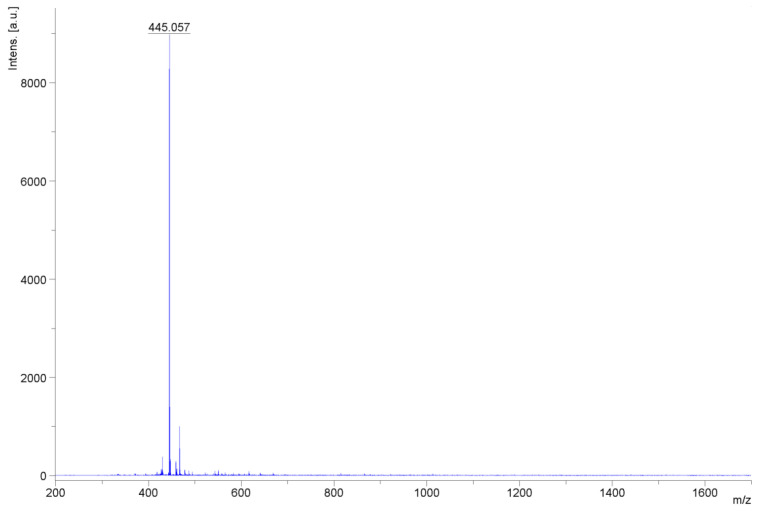
MALDI-TOF-MS spectra obtained for the monosodium salt (I).

The mass spectra recorded from the matrix-assisted laser desorption ionization-time-of-flight (MALDI-TOF) analysis showed that the main molecular species in MLES had a mass of 445.062 [M+Na]^+^, corresponding to the theoretical mass of mangiferin monosodium salt (Figure 5).

## 3. Discussion

The pharmacokinetic data gathered in this study indicate that, on average, the mangiferin in MLES is at least 2.4 times more bioavailable than that in MLE60 (*p* < 0.01). While further research is needed to fully understand the bioavailability of mangiferin—particularly regarding its absorption from herbal extracts or polyherbal formulations—we hypothesize that the increased bioavailability of mangiferin monosodium salt may primarily be attributed to its improved aqueous solubility [31].

In line with the above, a general strategy that has been employed on several occasions to enhance the absorption of flavonoids is based on increasing their hydrophilic nature [42]. The theory suggests that for flavonoids to be absorbed, they must first pass through an aqueous barrier (mucus) between the intestine and the epithelial cells, where they can be further deglycosylated to favor absorption or directly permeate the phospholipid bilayer of the cell membrane [30,43]. In the specific case of mangiferin, Tian and colleagues [22] found that the poor bioavailability of mangiferin can be attributed to its low solubility and amphipathic properties, which likely lead to diminished membrane permeability. These authors also state that mangiferin uptake may primarily depend on passive transport rather than uptake transporters or metabolic enzymes, such as uridine diphosphoglucuronosyltransferase (UGT) and cytochrome P450 (CYP450). In contrast, the inhibition of the transporter P-gp [23,24] increased the exposure of pure mangiferin by approximately threefold; however, this increase was not observed when the source of mangiferin was a decoction of Zhi-mu (*Anemarrhena asphodeloides*) or a mixture of mangiferin and timosaponin B2. This led the authors to conclude that the inhibition of P-gp is not a promising strategy for increasing mangiferin absorption.

In addition, the c-glycosyl bond present in mangiferin is more resistant to hydrolysis than the typical O-glycosidic bonds found in the majority of flavonoids, making it difficult to cleave the molecule into its monodeglycosylated metabolite, norathyriol. The formation of norathyriol is mediated by the enterobacterium *Bacteroides* sp. MANG [44], while its production via hepatic metabolism appears negligible [22]. However, this conversion is very slow (T_max_ is close to 15 h), and the absorption of norathyriol represents only a small fraction of the mangiferin dose (approximately 0.1%) [22,24].

To the best of our knowledge, Guo and co-workers [31] are the only authors who compared the bioavailability of mangiferin and its monosodium salt. In contrast to our work, these authors began with highly purified mangiferin (>98 %) of undescribed origin, from which they synthesized and purified the monosodium salt using water, acetone, and sodium bicarbonate (NaHCO_3_), also achieving a final purity of 98 %. Additionally, their pharmacokinetic study was conducted on forty male Sprague Dawley rats instead of humans. By comparing the relative bioavailability of mangiferin and its monosodium salt based on AUC_0–∞_, the authors reported that the absorption of the salt was 5.7 times greater than that of pure mangiferin. This finding significantly exceeds the relative bioavailability observed in our study for the salified *Mangifera indica* extract, which was 2.44-fold higher than that of the non-salified version. The authors also investigated the safety and toxicity of mangiferin monosodium salt in rats and mice. They determined that a dose of 400 mg of the purified salt was generally safe for rats, with no adverse effects reported. In mice, toxicity tests revealed a lethal dose (LD_50_) of 4000 mg/kg and a maximum tolerated dose (MTD) of 12,000 mg/kg, concluding that the salt was well tolerated and exhibited no signs of toxicity.

Finally, it is worth highlighting that the results obtained in our study are closely aligned with those reported by Hou and colleagues [19], who, to the best of our knowledge, have been the only ones to analyze the pharmacokinetics of mangiferin (>90% purity) in human plasma after oral administration. These authors found that the maximum concentrations of mangiferin were observed between 1 and 2 h (C_max_ of 19.94 ng/mL and 38.64 ng/mL at 100 mg and 900 mg oral doses, respectively), with half-lives ranging from 4.5 to 9 h. This study also confirmed that the absorption of mangiferin is non-linear; that is, the higher the dose, the lower the total absorption, although higher C_max_ values were observed with increasing doses.

## 4. Materials and Methods

### 4.1. Chemicals and Reagents

Reference standards of mangiferin and the internal standard (IS) were acquired from Chromadex (Irvine, CA, USA). Ultra-high-purity water was obtained using a Milli-Q water purification system. Methanol, acetonitrile, ammonium formate, and formic acid were of MS grade and sourced from Merck KGaA (Darmstadt, Germany). Additional reagents, including dimethyl sulfoxide (DMSO), deuterated DMSO-d6, n-butanol, and α-cyano-4-hydroxycinnamic acid, were also purchased from Merck KGaA (Darmstadt, Germany).

Mango leaf extracts (MLE60 and MLES) were obtained from Nektium Pharma, S.L. Maltodextrin was utilized as an excipient and procured from Comercial Química Massó, S.L. (Barcelona, Spain).

### 4.2. Plant Material

*Mangifera indica* leaves were collected in the Guanxi region (China), coinciding with the ripening and harvesting of the mango fruit in that area, from plants that had been cultivated for at least three years. The identity of the plant material was verified through DNA barcoding. The leaves were sun-dried for 3 to 5 days until the moisture content was below 12% (*w*/*w*) and then milled prior to high-temperature aqueous extraction. The mangiferin content in the dehydrated leaves ranged from 25 to 50 mg/g.

### 4.3. Mangifera Indica Leaf Extracts Preparations and Content of Mangiferin

Briefly, mango leaf extract standardized to 60% (*w*/*w*) mangiferin (MLE60) was obtained through serial aqueous extractions (×3) of dried and ground mango leaves at a high temperature (98 °C), followed by purification with affinity resins, forced precipitation via solvent evaporation, and spray drying. MLES was prepared from the MLE60 extract using an alkaline salification treatment. In this process, 2% (*w*/*w*) of MLE60 was dispersed in water heated to 80 °C. A sodium hydroxide solution (2 M) was then added to the mixture to achieve a pH of 8, and the contents were stirred for 15 min. Following this, the solution underwent spray-drying to create a powder containing approximately 60% (*w*/*w*) mangiferin monosodium salts. These salts were characterized using ^1^H and ^13^C-NMR and MALDI-TOF.

### 4.4. Preparation of Mangiferin Stock Solution and Quality Control Samples

Stock solutions of 0.5 mg/mL of mangiferin and the internal standard (IS) were prepared separately in a methanol/DMSO (4:1; *v*/*v*) mixture and in methanol, respectively. These two compounds were subsequently separately diluted into intermediate standard solutions in methanol at a concentration of 5 µg/mL. These intermediate standards were utilized to produce a series of working standard solutions prepared in acetonitrile containing 0.1% formic acid (*v*/*v*), which were ultimately employed to construct matrix-based calibration curves and quality control samples.

Matrix-based calibration samples were prepared daily by spiking 200 µL of blank human plasma with 400 µL of the working standard solutions. After complete sample pre-treatment (Section 4.6), each well was reconstituted in 100 µL of a methanol/water (1:1; *v*/*v*) mixture containing 20 mM of ammonium formate and 0.1% formic acid (*v*/*v*). The calibration curves consisted of a blank sample (a matrix sample without the internal standard), a zero sample (matrix samples containing the internal standard), and six calibration points ranging from 5 to 1000 ng/mL of mangiferin and 125 ng/mL of the internal standard.

Quality control (QC) samples were prepared in the same manner and evaluated at low, medium, and high concentrations: 50, 500, and 1000 ng/mL. Blank plasma samples were also included in the injection series to account for any potential carryover.

### 4.5. UHPLC-MS/MS Instrument and Conditions

An ultra-high-performance liquid chromatography (UHPLC) method with triple-quadrupole mass spectrometry (MS/MS) detection was employed to determine the levels of mangiferin in human plasma. Analyses were performed on an Acquity H-Class UPLC XEVO-TQD system (Waters, Milford, MA, USA). Separation was conducted on an Acquity C_18_ BEH column (50 × 2.1 mm, 1.7 µm). The mobile phases used were acetonitrile (A) and 0.1% (*v*/*v*) formic acid with 20 mM ammonium formate in water (B). The chromatographic gradient employed was as follows: from 0 to 0.5 min, 10% of A (isocratic); from 0.5 min to 1 min, 10% → 37.5% of A; from 1 min to 2 min, 37.5% of A (isocratic); from 2 min to 2.5 min, 37.5% → 95% of A; and from 2.5 min to 4.5 min, 95% → 10% of A (initial conditions). The flow rate was set to 0.4 mL/min, the column temperature to 40 °C, the sample vials to 12 °C, and the injection volume to 2 µL.

Multiple reaction monitoring (MRM) parameters were optimized by directly infusing each standard at 1 µg/mL in a methanol/water (2:1; *v*/*v*) mixture containing 20 mM ammonium formate and 0.1% formic acid. The standards were infused from 2 mL vials at a flow rate of 15 µL/min using a pump module located in the triple quadrupole detector.

Ionization within the ESI source was achieved using nitrogen as the nebulizing, cone, and drying gas. The desolvation and source temperatures were maintained at 150 °C and 500 °C, respectively. The desolvation and cone gas flow rates were set at 950 L/h and 50 L/h, respectively. The capillary voltage was fixed at 3.0 kV in negative mode (ESI^−^). The cone voltage was optimized for each compound. Collision-induced dissociation (CID) was conducted using argon as the collision gas at a fixed flow rate of 0.15 mL/min. The fragment ions obtained for each compound, along with the corresponding collision potentials, are presented in Table 5. The ions utilized for quantification and confirmation were monitored alongside the retention times for each analyte to ensure their presence in the samples.

### 4.6. Sample Preparation

#### 4.6.1. Instrumentation

Ostro^™^ plates were utilized for protein precipitation and phospholipid removal. A 96-well extraction plate vacuum manifold supported the Ostro^™^ plates. A Microvap 118 evaporator from Organomation (River Road, West Berlin, MA, USA) was employed for solvent evaporation under a gentle stream of nitrogen at a controlled temperature. An MM1500 series stirrer from LBX Instruments (Barcelona, Spain) was used to reconstitute the plasma samples prior to their injection into the UHPLC-MS/MS system.

#### 4.6.2. Plasma Samples

Before the chromatographic analysis, the plasma samples were thawed to room temperature. Then, 200 µL of plasma samples was pipetted into the wells of the Ostro^TM^ plates, which had been previously placed on a 2 mL collection plate. To induce protein precipitation, 400 µL of 0.1% formic acid in acetonitrile containing the IS (samples) or 400 µL of the working standard solutions (calibration points and QC samples) were added to the respective wells. Each well was thoroughly mixed by aspirating 10 times with the pipette immediately after the addition of the 400 µL acetonitrile solution. The samples were then vacuum-filtered through the Ostro™ wells to remove precipitated material and phospholipids. Each well was subsequently evaporated to dryness under a gentle stream of nitrogen at 50 °C. Before injection, each sample was reconstituted in 100 µL of a mixture of methanol and water (1:1, *v*/*v*) containing 0.1% formic acid and 20 mM ammonium formate, and placed into a 1.5 mL glass vial with 150 µL microvolume inserts.

### 4.7. Method Validation

The parameters evaluated included specificity, linearity, sensitivity, accuracy, precision, matrix effects, recovery, and stability.

#### 4.7.1. Specificity, Linearity and Sensitivity

Specificity was evaluated by comparing the following chromatograms: blank matrix samples, blank matrix samples spiked at the lowest concentration level of the calibration curve (including the IS), and samples collected during the pharmacokinetic assays (1 h after the oral administration of the treatments).

Linearity was determined by plotting the peak area ratios of mangiferin to the IS (*Y*-axis) against the nominal concentration of mangiferin (*X*-axis). A least squares linear regression model was used to fit the calibration curve (Y = A + BX), using 1/x as a weighting factor. Six calibration levels, with concentrations ranging from 5 to 1000 ng/mL, were prepared to evaluate this parameter. The lower limit of quantification (LLOQ) was defined as the lowest concentration on the calibration curve. The relative standard deviation (RSD, %) of a blank plasma sample spiked at the LLOQ must not exceed 20%. The acceptance criterion for accuracy is 100% ± 20%, based on six replicate analyses. Additionally, the signal-to-noise ratio at this level must be greater than 10.

#### 4.7.2. Accuracy and Precision

In the absence of certified reference material, method accuracy was determined as the mean percentage recovery from QC samples at three different concentration levels (n = 6). The accuracy of the assay was expressed as follows: calculated concentration/nominal concentration × 100%. Intra-day (repeatability) and inter-day (intermediate) precision were evaluated at the same levels used in the accuracy analysis (n = 6). Analyses were performed on the same day (intra-day) and on two different days by two analysts (inter-day). The acceptance criterion for accuracy is set at 80–120%. Precision was required to be within a relative standard deviation (RSD) of 15%, except for the lower limit of quantification (LLOQ), which was required to be within 20%.

#### 4.7.3. Matrix Effect, Recovery and Stability

QC samples at three different levels (n = 6) were analyzed to calculate the matrix effect and extraction recovery. The matrix effect was analyzed by directly comparing the mangiferin peak area in post-extraction spiked matrix samples to that obtained from pure standard solutions. The extraction recoveries were determined by comparing the peak areas of mangiferin in pre-extraction spiked matrix samples, following the application of the entire sample preparation protocol, to those obtained from pure mangiferin standard solutions at the same concentration levels.

The stability of mangiferin in human plasma was evaluated by analyzing the same three levels of QC samples (n = 6) after storage at room temperature for 4 h, in the autosampler for 4 h at 12 °C, and after three cycles of freezing (−80 °C) and thawing (20 °C).

### 4.8. Pharmacokinetic Protocol

MLE60 and MLES were orally administered to 12 healthy volunteers (6 men and 6 women, Table 6), who were selected according to the following criteria:

- Over 18 years old and with a normal body mass index (BMI) (<30), non-smokers;
- The subjects were determined to be healthy based on their medical history: normal liver and renal functions, without food allergies;
- No drug intake was allowed 2 weeks prior to or during this study;
- The subjects were asked not to consume meals containing mango fruit or mango leaf tea for three days before this study;
- Females were required to have regular menstrual cycles without the use of oral contraceptives.

**Figure 6:**
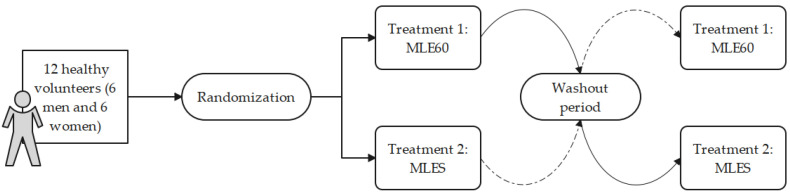
Pharmacokinetic study design (n = 12; crossover), including randomization and washout period (7 days).

The participants ingested both formulations on different days (crossover) following an overnight fast of at least 12 h, with a minimum washout period of 7 days between the treatments (Figure 6). The intake of each formulation was randomized before the start of this study. A total of 783.5 mg of MLE60 (61.24% mangiferin, *w*/*w*) and 805.3 mg of MLES (59.65% mangiferin, *w*/*w*) were dispensed orally to each subject. The two formulations were administered with 250 mL of water, and the subjects were restricted from consuming any food for the first four hours post-administration. After a blood sample was taken at the fourth hour, the participants were allowed to resume their normal meals.

Blood samples (5 mL each) were collected using cannulas into heparinized tubes at predetermined time points: 0 h (prior to extract administration), 0.25 h, 0.5 h, 1 h, 2 h, 4 h, 6 h, 10 h, and 24 h post-administration. Plasma was separated by centrifugation at 3000 rpm for 5 minutes and stored at −80 °C for future analysis, resulting in nine samples per participant. Additional blank plasma samples were collected following the same procedure and utilized to construct daily calibration curves. Pharmacokinetic curves were generated by analyzing mangiferin concentrations in plasma samples at each collection time using the UHPLC-MS/MS method described above.

### 4.9. Analysis of Pharmacokinetic Data

Noncompartmental analysis (NCA) using the linear trapezoidal rule was employed to calculate pharmacokinetic parameters with the PKNCA package (v.0.11.0) [45] implemented in R [46]. Mangiferin concentrations in plasma samples were normalized according to the administered dose of mangiferin and the body weight of each participant [47].

Dose-normalized concentrations (DNCs) were calculated using the following equation:

$$DNC=\frac{C}{\left(D/BW\right)}$$

where:

- C: concentration of mangiferin in the plasma samples in ng/mL;
- D: dose of mangiferin (479.8 mg for MLE60, 480.4 mg for MLES);
- BW: body weight in kg.

The Shapiro–Wilk test was used to compare the normality of the datasets, and the Wilcoxon–Mann–Whitney test was employed to compare dose-normalized areas under the curve (AUCs).

### 4.10. NMR and MALDI-TOF Confirmation Analysis

The formation of the monosodium salt of mangiferin in the MLES extract was confirmed by ^1^H-NMR, ^13^C-NMR and MALDI-TOF mass spectrometry analysis. In summary, a 500 MHz Plus Avance II NMR (Bruker, Switzerland) was used for nuclear magnetic resonance analyses. ^1^H-NMR spectra were recorded at 500 MHz at room temperature (21 °C). The samples (15–20 mg) were dissolved in deuterated dimethyl sulfoxide (DMSO-d_6_, 0.75 mL), with residual solvent peaks at δ = 2.50 (q, DMSO) and δ = 3.34 (br, H_2_O) ppm for ^1^H and δ = 40.0 (DMSO) for ^13^C.

Mangiferin ^1^H NMR (500 MHz, DMSO) δ 13.77 (s, 1H, C-1-OH), 10.43 (s, 3H, C-3-OH, C-6-OH, C-7-OH), 7.38 (s, 1H, H-8), 6.86 (s, 1H, H-5), 6.37 (s, 1H, H-4), 4.86 (br, 2H), 4.59 (d, J = 9.8 Hz, 1H, glc-H-1), 4.48 (br, 2H), 4.05 (t, J = 9.3 Hz, 1H, glc-H-2), 3.69 (d, J = 11.9,1H, glc-H-6), 3.41 (dd, J = 11.8, 6.0 Hz, 1H, glc-H-6), 3.24–3.09 (m, 3H, glc-H-4, glc-H-5, glc-H-3).^13^C NMR (126 MHz, DMSO) δ 179.1 (C-9), 163.8 (C-3), 161.8 (C-1), 156.2 (C-4a), 154.2 (C-6), 150.8 (C-10a), 143.8 (C-7), 111.6 (C-8a), 107.9 (C-8), 107.6 (C-2), 102.6 (C-5), 101.3 (C-8b), 93.3 (C-4), 81.6 (glc-5), 79.0 (glc-3), 73.0 (glc-1), 70.6 (glc-2), 70.2 (glc-4), 61.5 (glc-6).Exact. Mass: 421.077 (M-H)^−^.

Monosodium mangiferin ^1^H NMR (500 MHz, DMSO) δ 14.71 (s, 1H, C-1-OH), 6.89 (s, 1H, H-8), 5.98 (s, 1H, H-5), 5.96 (s, 1H, H-4), 4.56 (d, J = 9.5 Hz, 1H, glc-H-1), 4.45 (br, 6H), 4.12 (t, J = 9.5 Hz, 1H, glc-H-2), 3.63 (dd, J = 11.6, 2.4 Hz, 1H, glc-H-6), 3.52 (dd, J = 11.6, 4.8 Hz, 1H, glc-H-6), 3.32–3.18 (m, 2H, glc-H-3, glc-H-4), 3.14 (ddd, J = 9.4, 4.8, 2.4 Hz, 1H, glc-H-5). ^13^C NMR (126 MHz, DMSO) δ 176.8 (C-9), 168.1 (C-6), 162,4 (C-3), 162.1 (C-2), 156.6 (C-4a), 155.0 (C-4b), 147.5 (C-7), 107.4 (C-2), 103.4 (C-8), 100.6 (C-8b), 99.0 (C-5), 94.4 (C-4), 86.3 (C-8a), 81.4 (glc-5), 79.7 (glc-3), 74.5 (glc-1), 70.7 (glc-4), 70.6 (glc-2), 61.2 (glc-6). MALTI-TOF: 445.062 [M+Na]^+^.

The dried droplet method was used to prepare the sample for MALDI analysis. Briefly, the samples were mixed in an Eppendorf tube at a 1:5 ratio with the 2,5-Dihydroxybenzoic acid (DHB) matrix. The matrix was prepared at a concentration of 15 mg/mL and dissolved in TA50 (50%; *v*/*v*) acetonitrile and 0.1% (*v*/*v*) trifluoroacetic acid in distilled water. Then, a 2 µL volume of the sample–matrix mixture was spotted on a stainless-steel sample plate and allowed to dry for 10 min at room temperature. Mass spectra were recorded with an ultrafleXtreme^®^ MALDI-TOF mass spectrometer (Bruker Daltonics, Billerica, MA, USA) equipped with a nitrogen laser emitting at 337 nm and operated in reflectron positive mode using the flexControl software (version 3.4; Bruker Daltonics). The laser power was manually adjusted until the optimum signal-to-noise ratio was obtained, ensuring that each acquired spectrum resulted from the accumulation of a minimum of 2500 laser shots. Spectra were analyzed using the Flex Analysis software (flexControl 3.4.135.0, Bruker Daltonics).

#### Samples Preparation

For the isolation of mangiferin from MLE60, 500 mg of extract was dissolved in n-BuOH/water (1:1, 0.5 mL) and subjected to reverse-phase filtration on RP-18 eluting with a gradient of H_2_O and MeOH with a stepwise increasing amount of MeOH. The mangiferin fraction was then collected and concentrated to dryness to obtain the purified molecule. The isolation of mangiferin monosodium salts from MLES was achieved by dissolving the extract in a mixture of acetone/water 100 mL (3:1), which was kept at 0 °C for 5 h. After this time, the precipitate obtained was filtered under vacuum and characterized by NMR analysis and MALDI-TOF mass spectrometry.

## 5. Conclusions

In this study, a simple, rapid, sensitive, and high-throughput UHPLC-ESI-MS/MS method was validated and applied to quantify mangiferin in human plasma. The use of Ostro™ pass-through plates effectively removed phospholipids from plasma samples while enabling in-well protein precipitation, analyte extraction from plasma into the selected solvent, and particulate filtration. This approach streamlined sample preparation procedures and enhanced the precision and sensitivity of the method.

The pharmacokinetic data obtained in this study demonstrate that converting mangiferin into its monosodium salt derivative significantly improves the bioavailability of this compound. When comparing the AUC_0–24h_ of the two treatments (n = 12), the absorption of mangiferin was 2.44 times higher in MLES (*p* = 0.0029 **). In addition, when dose-normalized AUC_0–t_ (n = 12) was analyzed per time point, it was observed that the differences in the absorption of mangiferin were greater within the first two hours after the ingestion of the extracts: 3.19 times more from MLES than from MLE60 (*p* < 0.01 **) after 1 h and 3.24 times higher after 2 h (*p* < 0.01 **). This suggests that mangiferin absorption from MLES is faster and more acute than that from MLE60.

We hypothesized in this study that the increased solubility of mangiferin monosodium salts compared to the unprocessed xanthonoid may lead to enhanced intestinal permeability and absorption. Evaluation of the behavior of mangiferin and its monosodium salts in in vitro gastric models may help clarify the mechanisms underlying the improved absorption of MLES observed in this study and will be the focus of future investigations. Furthermore, given the reversible nature of the sodium salts and the knowledge that their dissociation is favored in acidic solutions, it could also be hypothesized that if we had omitted the gastric step (e.g., by targeted encapsulation) or buffered the pH in the stomach (e.g., by using proteins in the final formulation), the differences in absorption between these two treatments could have been greater.

Considering all the above, the salification approach evaluated in this study offers a straightforward strategy for producing standardized mango leaf extracts with enhanced health-promoting benefits. It also extends the range of applications in which the extract can be incorporated into beverages and other products where solubility may be a limiting factor for commercialization.

## Figures and Tables

**Table 1:** Precision and accuracy in the determination of mangiferin in human plasma samples (n = 6).

Compound	Nominal Concentration (ng/mL)	Accuracy		Precision	
		Calculated Concentration (ng/mL) ± SD	Accuracy (%)	Intra-Day (RSD, %)	Inter-Day (RSD, %)
Mangiferin	5 (LLOQ)	4.79 ± 0.40	95.74	8.28	-
	50	51.11 ± 6.09	102.22	11.91	9.47
	500	517.83 ± 32.06	103.57	6.19	5.85
	1000	979.73 ± 24.34	97.93	2.48	2.05

**Table 2:** Extraction recovery, matrix effects, and stability of mangiferin in human plasma quality control samples (n = 6).

Compound	Nominal Concentration (ng/mL)	Recovery (%)		Matrix Effect (%)		Stability (%, Mean ± RSD)		
		Average	RSD	Average	RSD	Room Temp.	Autosampler	Freeze–Thaw
Mangiferin	50	74.41	12.42	96.51	11.44	103.10 ± 3.66	109.92 ± 2.77	108.60 ± 3.87
	500	74.90	4.18	109.99	3.60	101.27 ± 9.08	102.02 ± 8.81	101.68 ± 0.91
	1.000	81.11	4.66	106.48	6.12	101.75 ± 8.11	101.63 ± 8.50	102.64 ± 1.05

**Table 3:** Comparison of pharmacokinetic parameters after treatment administration (n = 12).

Parameter ^1^	Units	MLE60	MLES
AUC_0–1h_	h x ((ng/mL)/(mg/kg))	2.16 [81.5]	6.88 [51.4]
AUC_0–2h_	h x ((ng/mL)/(mg/kg))	6.35 [75.7]	20.6 [46.8]
AUC_0–4h_	h x ((ng/mL)/(mg/kg))	14.1 [64.5]	39.2 [53.3]
AUC_0–6h_	h x ((ng/mL)/(mg/kg))	18.4 [72.7]	47.3 [57.7]
AUC_0–10h_	h x ((ng/mL)/(mg/kg))	21.9 [83.8]	53.3 [63.1]
AUC_0–24h_	h x ((ng/mL)/(mg/kg))	22.7 [87.8]	55.5 [64.5]
AUC_0–∞_	h x ((ng/mL)/(mg/kg))	24.9 [97.8]	59.7 [68.4]
C_max_	ng/mL	38.8 [41.1]	103 [41.6]
T_max_	h	2.00 [1.00, 4.00]	2.00 [1.00, 2.00]
T_1/2_	h	3.19 [1.68]	2.98 [2.04]
V_z_/F	L/kg	157.0 [46.4]	58 [72.1]
Cl/F	(L/h)/kg	40.1 [97.8]	16.7 [68.4]

^1^ AUC_0–t_, C_max_, V_z_/F and Cl/F: geometric mean and geometric coefficient of variation; T_max_: median and range; half-life (T_1/2_): arithmetic mean and standard deviation.

**Table 4:** Bioequivalence comparison of the absorption of mangiferin from MLES and MLE0 for pairwise samples (n = 12). Significance level: ** < 0.01.

	0–1 h	0–2 h	0–4 h	0–6 h	0–10 h	0–24 h
**MLES vs. MLE60**	3.19 **	3.24 **	2.78 **	2.57 **	2.43 **	2.44 **

**Table 5:** Tandem mass spectrometry parameters utilized for the determination of mangiferin and the internal standard (IS).

Compound	Precursor Ion (m/z)	Product Ion [Collision Energy (eV)]		Cone Voltage (V)	Ion Mode
		Quantification Ion (m/z)	Qualification Ions (m/z)		
Mangiferin	421.0	330.0 [24]	300.9 [22]; 271.7 [36]	48	ESI^−^
Taxifolin (IS)	303.0	285.0 [10]	176.9 [10]; 124.9 [20]	42	ESI^−^

**Table 6:** Subjects included in this study, randomization of treatments, and doses received. The average weight (arithmetic mean) of males and females was 83.47 kg and 61.62 kg, respectively.

Subject Number	Male = M, Female = F	Body Weight (kg)	Experiment Day (P1 or P2)	Treatment	Mangiferin Dose (mg)	Normalized Dose (mg/kg BW)
1	M	80.7	P1	MLE60	479.80	5.95
		80.6	P2	MLES	480.38	5.96
2	F	61.5	P1	MLE60	479.80	7.80
		60.7	P2	MLES	480.38	7.91
3	F	61.4	P1	MLE60	479.80	7.81
		61.2	P2	MLES	480.38	7.85
4	M	86.4	P1	MLES	480.38	5.56
		84.7	P2	MLE60	479.80	5.67
5	M	83.6	P1	MLES	480.38	5.75
		82.7	P2	MLE60	479.80	5.80
6	F	62.9	P1	MLES	480.38	7.64
		62.6	P2	MLE60	479.80	7.67
7	F	63.7	P1	MLES	480.38	7.54
		63.6	P2	MLE60	479.80	7.54
8	F	48.7	P1	MLE60	479.80	9.85
		48.3	P2	MLES	480.38	9.95
9	M	88.2	P1	MLE60	479.80	5.44
		88.6	P2	MLES	480.38	5.42
10	F	B72	P1	MLE60	479.80	6.66
		72.8	P2	MLES	480.38	6.60
11	M	78.9	P1	MLES	480.38	6.09
		79	P2	MLE60	479.80	6.07
12	M	83.3	P1	MLES	480.38	5.77
		84.9	P2	MLE60	479.80	5.65

## Data Availability

The data supporting the conclusions of this article are available within the manuscript and the Supplementary Materials. Additional information can be obtained from the corresponding author (tvega@nektium.com). Deidentified participant data are also accessible upon reasonable request for research purposes.

## Supplementary Materials

The following supporting information can be downloaded at: https://www.mdpi.com/article/10.3390/molecules30030461/s1. Figure S1: Comparison of a UHPLC-DAD chromatogram obtained at 258 nm (a), and a UHPLC-MSMS TIC chromatogram (b), both taken from a blank plasma sample spiked at 500 ng/mL mangiferin; Figure S2: Individual pharmacokinetic curves obtained for each of the twelve subjects included in the study. Figure S3: Wilcoxon–Mann–Whitney box-and-whisker plots on pairwise samples: comparisons of AUC_0–24_ by sex and treatment. Figure S4: ^1^H-NMR and ^13^C-NMR spectrum. Figure S5: Exact mass for mangiferin by HPLC-MS. Table S1: Mangiferin plasma concentrations and standard deviations per subject (n = 12) and sampling time.
