# Supplementary material for: Human Pharmacokinetic Profiling and Comparative Analysis of Mangiferin and Its Monosodium Derivative from Mangifera indica Extracts Using UHPLC-MS/MS with 1H NMR and MALDI-TOF Confirmation
Source: Molecules. 2025 Jan 21;30(3):461. doi: 10.3390/molecules30030461 (PMC11820055; doi:10.3390/molecules30030461)
Supplement: Supplementary file 1 [file molecules-30-00461-s001.zip › molecules-3409790-supplementary.pdf]

Supplementary Information

Human Pharmacokinetic Profiling and Comparative Analysis of Mangiferin and Its Monosodium Derivative from *Mangifera indica* Extracts Using UHPLC-MS/MS with ^{1}H NMR and MALDI-TOF Confirmation

David Fuentes-Rios[1,2,] Alvaro Sanchez-Rodriguez[1], Laura Lopez-Rios[1], Eduardo Garcia-Gonzalez[3], Miriam Martinez-Canton[3], Victor Galvan-Alvarez[3], Angel Gallego-Selles[3], Marcos Martin-Rincon[3], Jose A.L. Calbet[3], Tanausu Vega-Morales[1,4,*]

1 Nektium Pharma SL, C/Las Mimosas 8, Polígono Industrial Arinaga, Las Palmas, 35118; dfuentes@nektium.com (D.F.R.); asanchez@nektium.com (A.S.R.); llopez@nektium.com (L.L.R.); tvega@nektium.com (T.V.M.)

2 Department of Organic Chemistry, Faculty of Sciences, University of Malaga, Campus de Teatinos s/n, 29071 Málaga, Spain

3 Department of Physical Education and Research Institute of Biomedical and Health Sciences (IUIBS), University of Las Palmas de Gran Canaria, Campus Universitario de Tafira s/n, 35017, Las Palmas de Gran Canaria, Spain; eduardo.garcia124@alu.ulpgc.es (E.G.G.); miriammartinezcanton@gmail.com (M.M.C.); victor_galvan@hotmail.es (V.G.A.); victor_galvan@hotmail.es (V.G.A.); angelgallegoselles@hotmail.com (A.G.-S.); marcos.martinrincon@gmail.com (M.M.R.); lopezcalbet@gmail.com (J.A.L.C.)

4 Instituto Universitario de Estudios Ambientales y Recursos Naturales (i-UNAT), University of Las Palmas de Gran Canaria, Campus Universitario de Tafira s/n, 35017, Las Palmas de Gran Canaria

* Correspondence: tvega@nektium.com; Tel.: +34 928 734 132

List of supplementary materials

1. Figure S1: Mangiferin specificity_MSMS vs PDA retention times and spectra.

2. Figure S2: Pharmacokinetic curves obtained per subject (n=12).

3. Figure S3: Wilconxon-Mann-Whitney box-and-whisker plot_pairwise_sex variable.

4. Figure S4: ^{1}H-NMR and ^{13}C-NMR spectrum.

5. Figure S5. Exact Mass for Magniferin by HPLC-MS.

6. Table S1: Mangiferin plasma concentrations.

Figure S1. Mangiferin specifcity_MSMS vs PDA retention times and spectra.

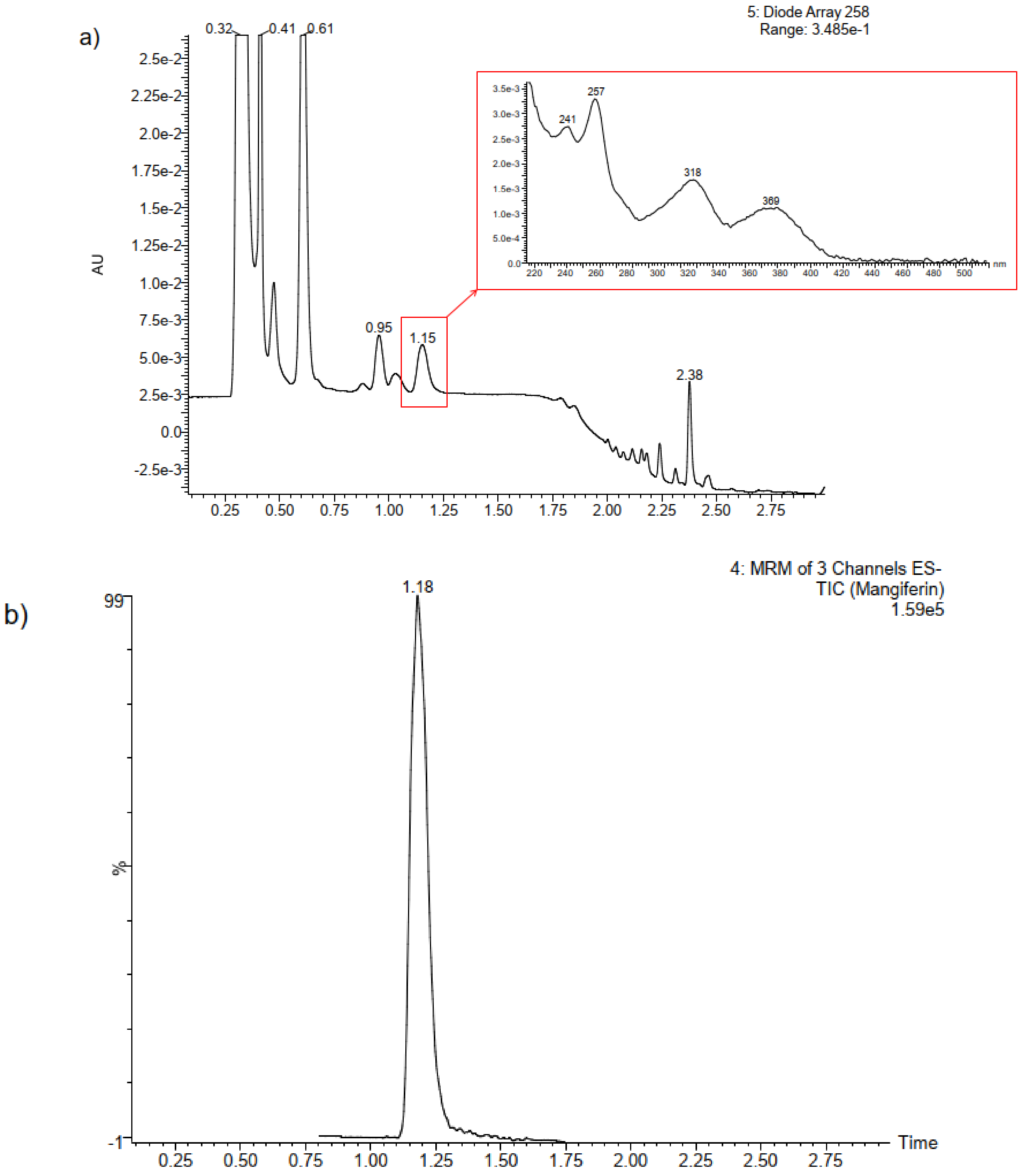

Figure S2. Pharmacokinetic curves obtained per subject (n=12).

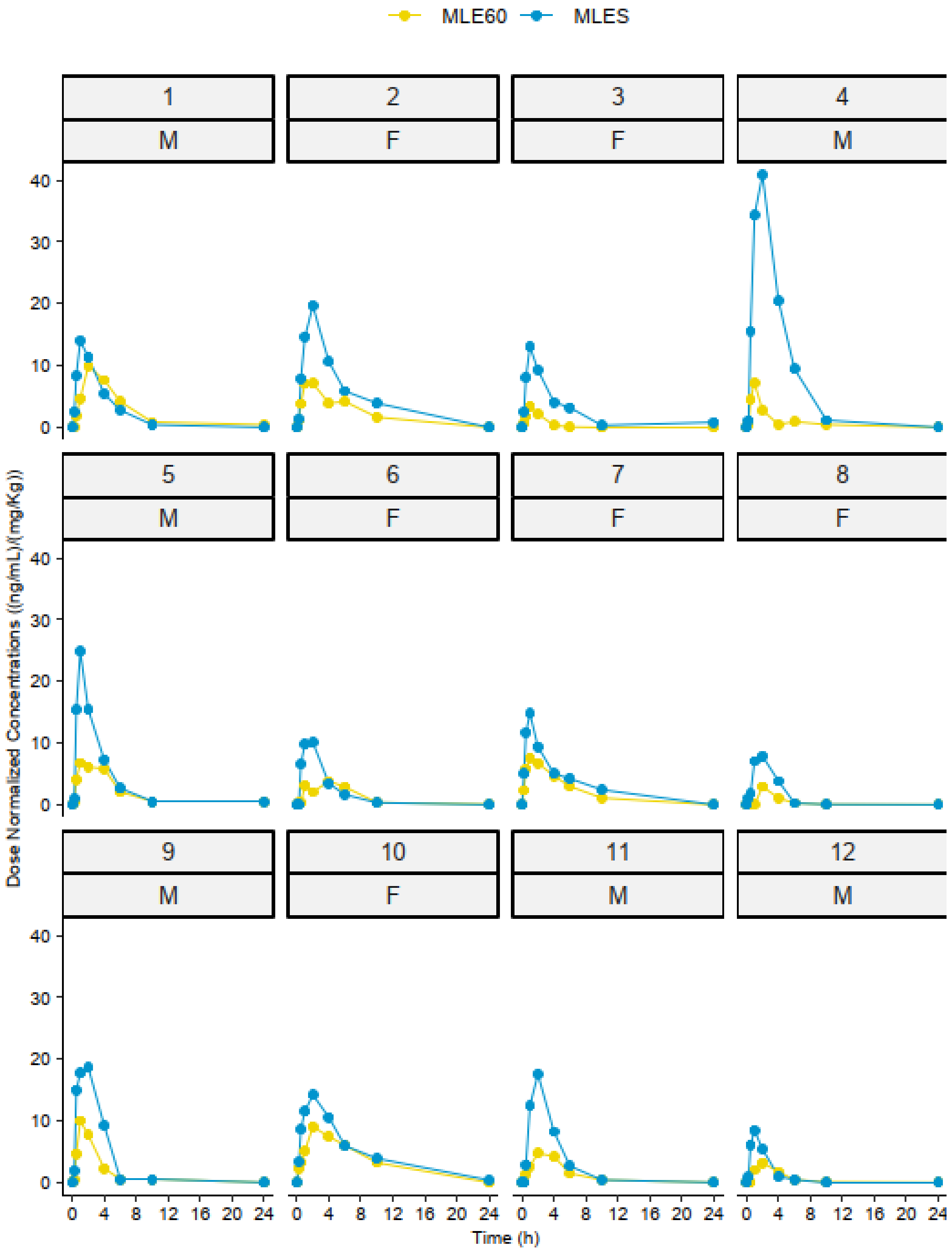

Figure S3. Wilconxon-Mann-Whitney box-and-whisker plot_pairwise_sex variable.

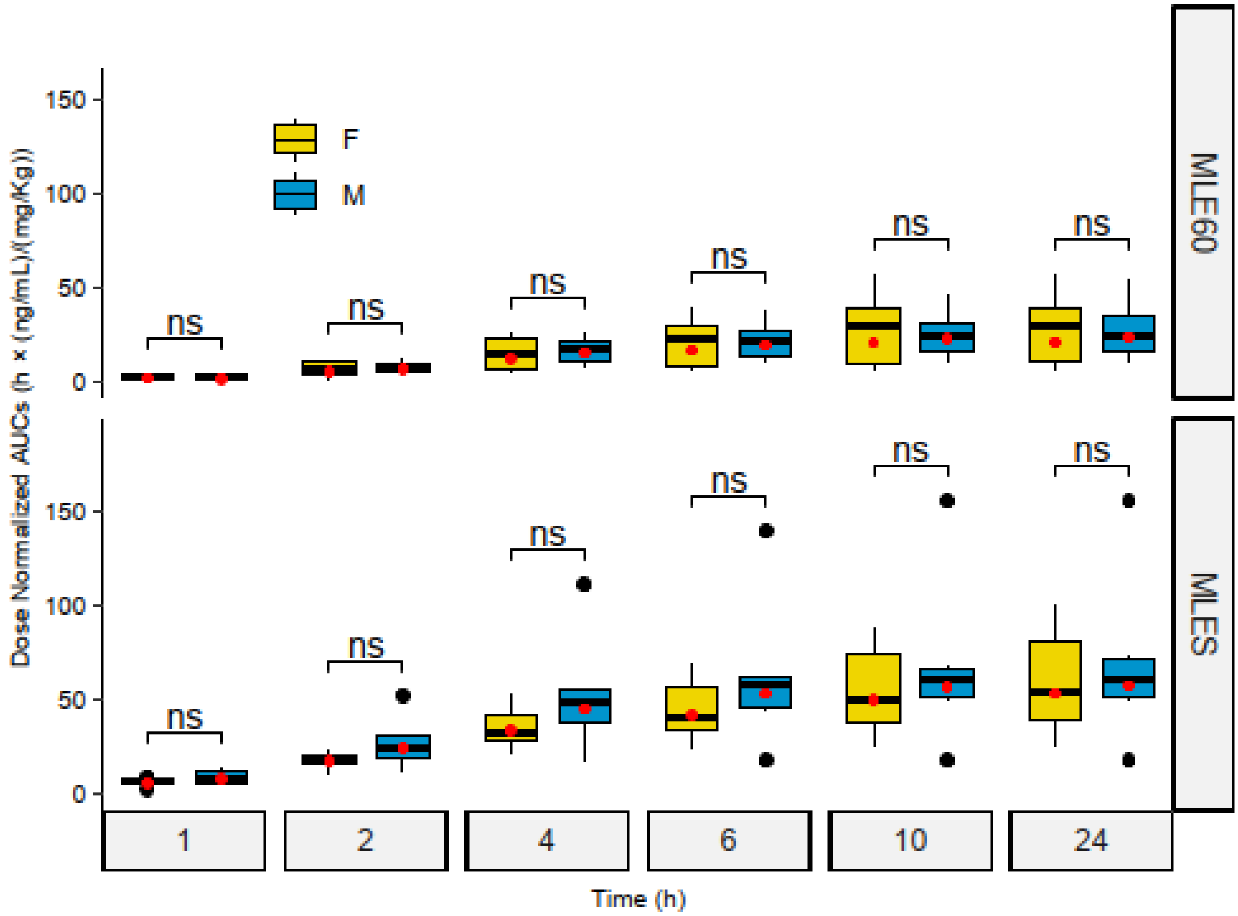

Figure S4. **^{1}H-NMR and ^{13}C-NMR spectrum of Mangiferin and its monosodium derivative.**

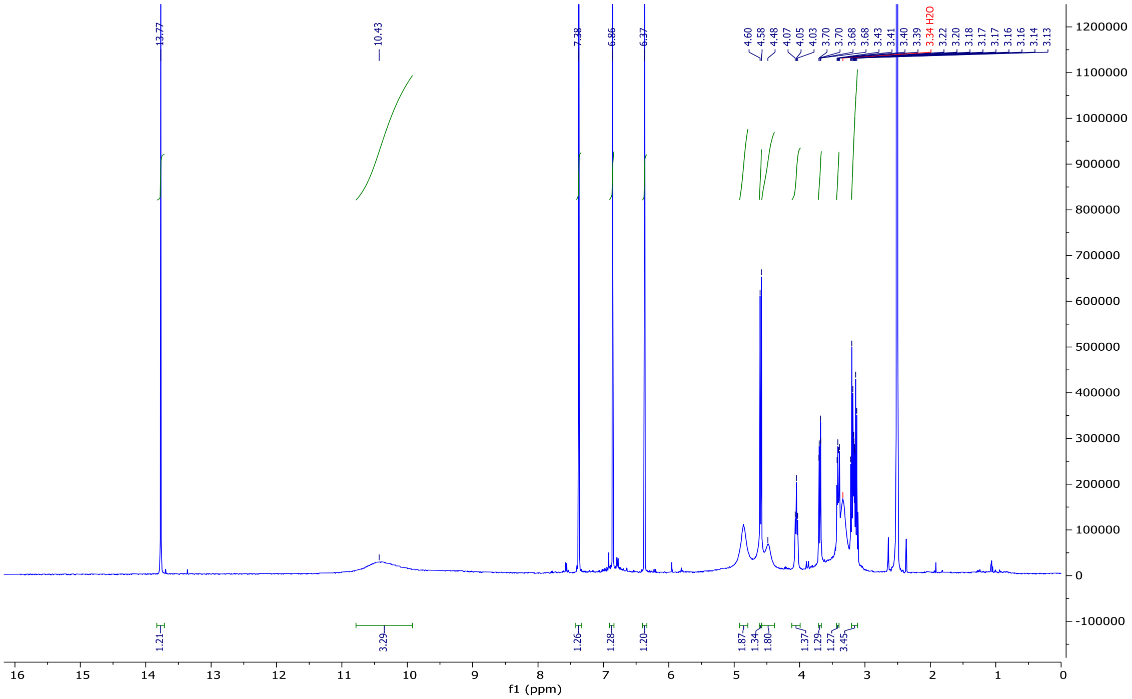

Mangiferin - ^{1}H NMR (500 MHz, DMSO) δ 13.77 (s, 1H, C-1-OH), 10.43 (s, 3H, C-3-OH, C-6-OH, C-7-OH), 7.38 (s, 1H, H-8), 6.86 (s, 1H, H-5), 6.37 (s, 1H, H-4), 4.86 (br, 2H), 4.59 (d, *J* = 9.8 Hz, 1H, glc-H-1), 4.48 (br, 2H), 4.05 (t, *J* = 9.3 Hz, 1H, glc-H-2), 3.69 (d, J = 11.9,1H, glc-H-6), 3.41 (dd, *J* = 11.8, 6.0 Hz, 1H, glc-H-6), 3.24 – 3.09 (m, 3H, glc-H-4, glc-H-5, glc-H-3).

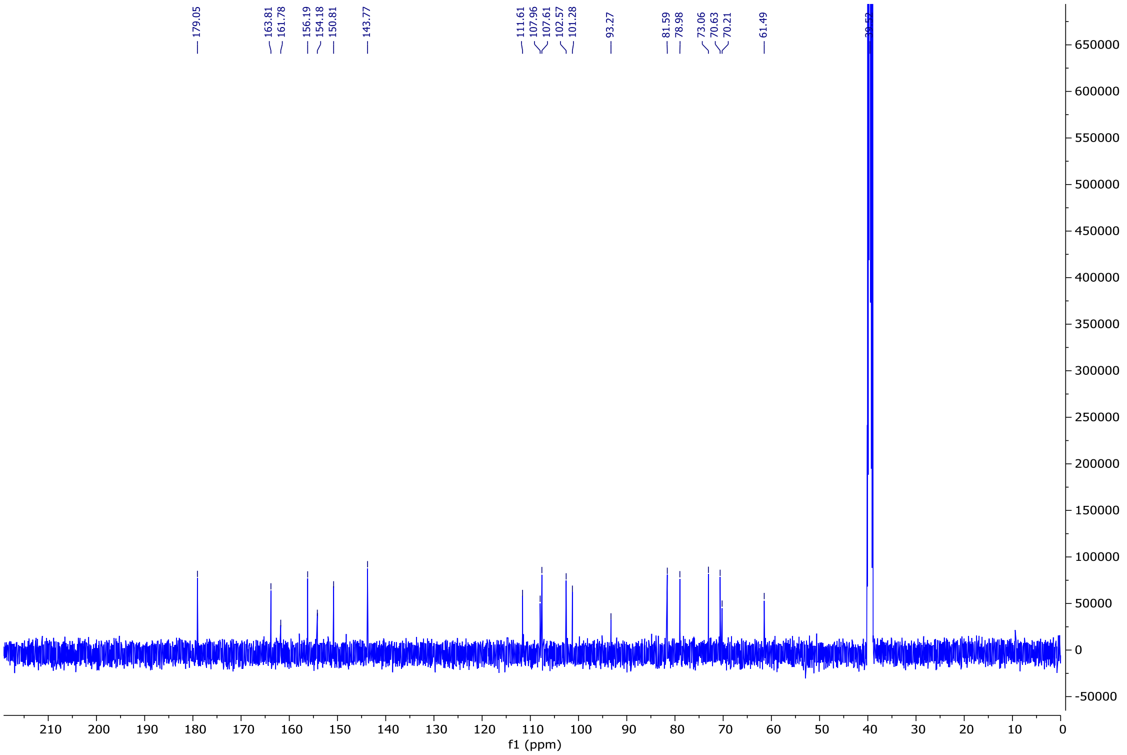

¹³C NMR (126 MHz, DMSO) δ 179.1 (C-9), 163.8 (C-3), 161.8 (C-1), 156.2 (C-4a), 154.2 (C-6), 150.8 (C-4b), 143.8 (C-7), 111.6 (C-8a), 107.9 (C-8), 107.6 (C-2), 102.6 (C-5), 101.3 (C-8b), 93.3 (C-4), 81.6 (glc-5), 79.0 (glc-3), 73.0 (glc-1), 70.6 (glc-2), 70.2 (glc-4), 61.5 (glc-6).

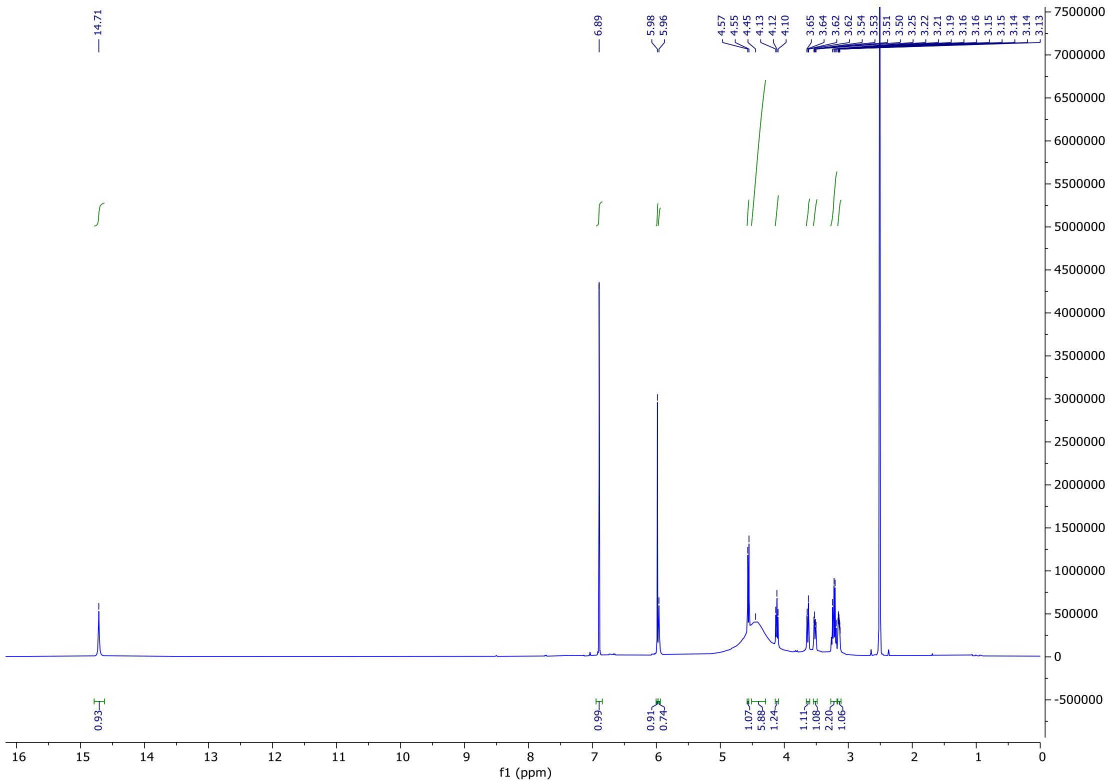

Monosodium derivative Mangiferin - ¹H NMR (500 MHz, DMSO) δ 14.71 (s, 1H, C-1-OH), 6.89 (s, 1H, H-8), 5.98 (s, 1H, H-5), 5.96 (s, 1H, H-4), 4.56 (d, *J* = 9.5 Hz, 1H, glc-H-1), 4.45 (br, 6H), 4.12 (t, *J* = 9.5 Hz, 1H, glc-H-2), 3.63 (dd, *J* = 11.6, 2.4 Hz, 1H, glc-H-6), 3.52 (dd, *J* = 11.6, 4.8 Hz, 1H, glc-H-6), 3.32 – 3.18 (m, 2H, glc-H-ç3, glc-H-4), 3.14 (ddd, *J* = 9.4, 4.8, 2.4 Hz, 1H, glc-H-5).

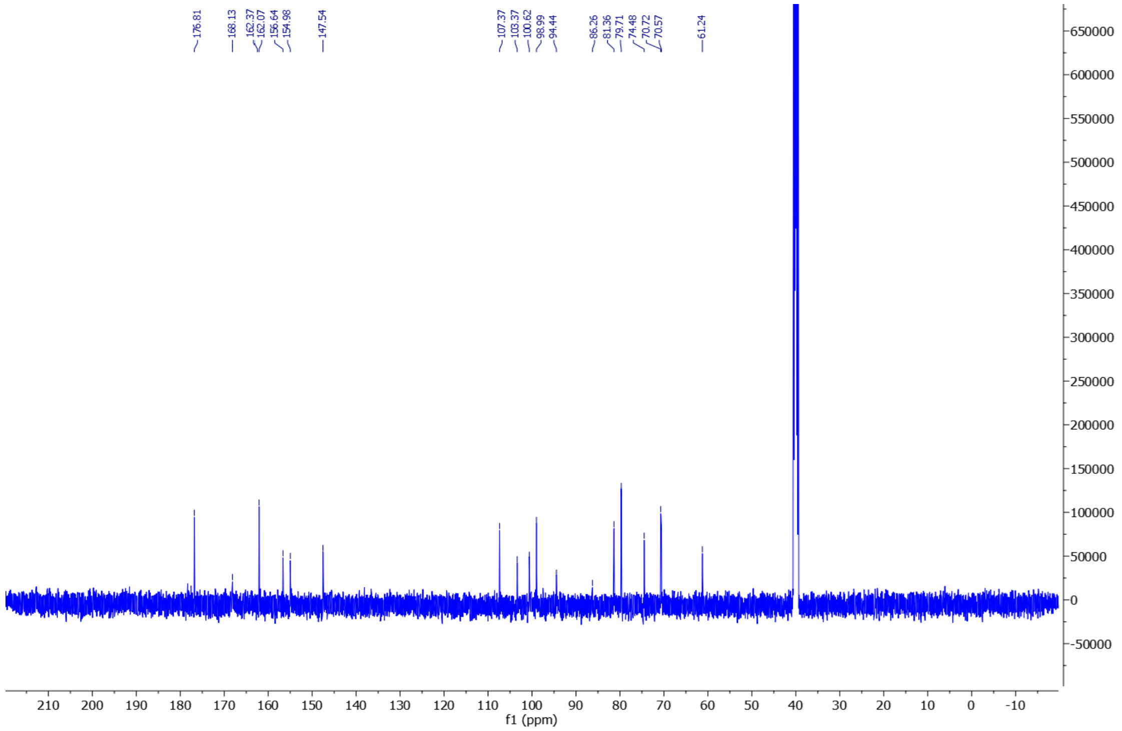

^{13}C NMR (126 MHz, DMSO) δ 176.8 (C-9), 168.1 (C-6), 162,4 (C-3), 162.1 (C-2), 156.6 (C-4a), 155.0 (C-4b), 147.5 (C-7), 107.4 (C-2), 103.4 (C-8), 100.6 (C-8b), 99.0 (C-5), 94.4 (C-4), 86.3 (C-8a), 81.4 (glc-5), 79.7 (glc-3), 74.5 (glc-1), 70.7 (glc-4), 70.6 (glc-2), 61.2 (glc-6).

Figure S5: Exact Mass for Mangiferin by HPLC-MS.

Exact Mass: 421.077

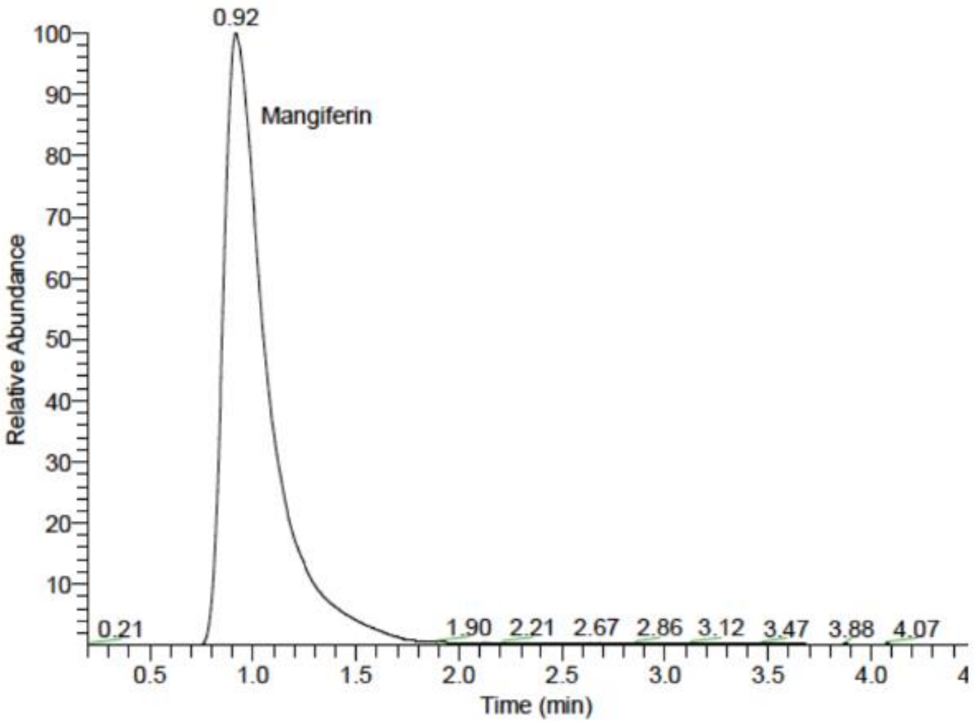

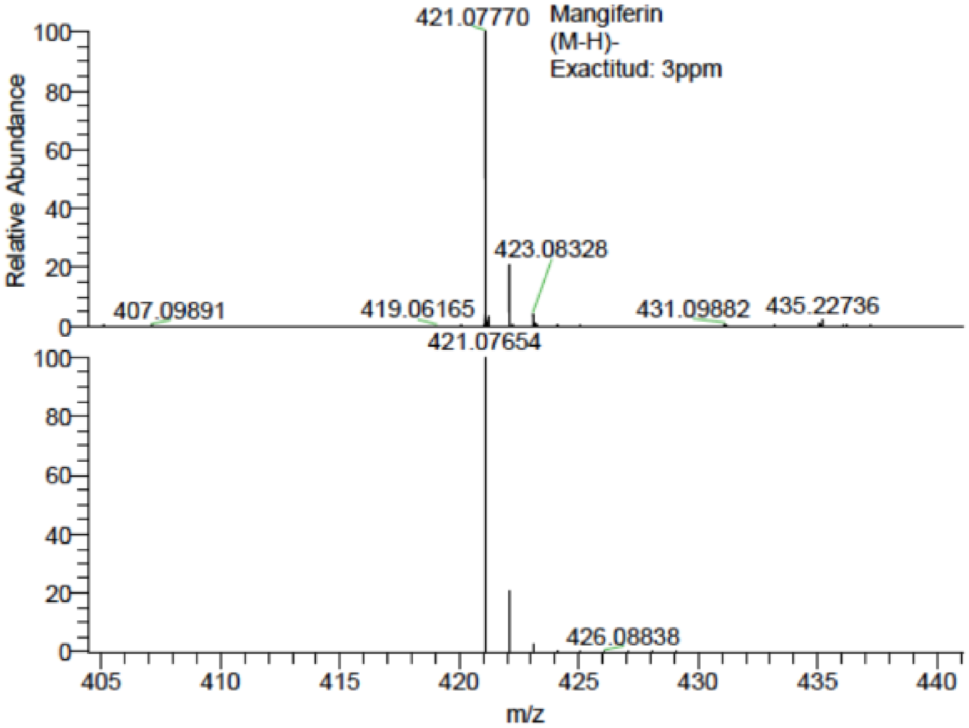

Table S1. Mangiferin concentrations (ng/L) and standard deviations in plasma samples from each subject, and at each sampling time.

Subject	Time (h)	Conc. (ng / mL)	Desv. Std.	Treatment	Sex
	0	0	0		M
	0.25	0	0		M
	0.5	10.99	0.827		M
	1	27.85	1.828		M
1	2	59.19	17.997	MLE60	M
	4	45.56	6.045		M
	6	24.81	5.69		M
	10	5.14	0.866		M
	24	<LLOQ[1]	-		M
	0	0	0		M
	0.25	15.01	2.243		M
	0.5	49.92	1.666		M
	1	83.81	4.449		M
1	2	67.74	4.128	MLES	M
	4	32.49	1.932		M
	6	16.62	3.137		M
	10	<LLOQ[1]	-		M
	24	0	0		M
	0	0	0		F
	0.25	9.07	1.901		F
	0.5	29.94	8.824		F
	1	55.3	9.978		F
2	2	55.9	1.365	MLE60	F
	4	30.43	3.046		F
	6	32.66	3.529		F
	10	12.85	1.505		F
	24	0	0		F
	0	0	0		F
	0.25	10.76	0.466		F
	0.5	62.52	1.788		F
	1	116.17	5.074		F
2	2	155.49	11.204	MLES	F
	4	84.19	9.563		F
	6	46.54	5.4		F
	10	31.04	5.855		F
	24	0	0		F
	0	0	0		F
	0.25	5.2	2.042		F
3	0.5	13.65	0.492	MLE60	F
	1	26.6	2.919		F
	2	17.08	3.152		F

	4	<LLOQ[1]	-		F
	6	<LLOQ[1]	-		F
	10	0	0		F
	24	0	0		F
3	0	0	0	ZYN Salt	F
3	0.25	19.8	3.915	ZYN Salt	F
3	0.5	63.62	7.971	ZYN Salt	F
3	1	102.77	7.945	ZYN Salt	F
3	2	73.13	6.605	ZYN Salt	F
3	4	32.05	4.927	ZYN Salt	F
3	6	25.19	0,16	ZYN Salt	F
3	10	<LLOQ[1]	-	ZYN Salt	F
3	24	6.42	1.547	ZYN Salt	F
4	0	0	0	ZYN	M
4	0,25	<LLOQ[1]	-	ZYN	M
4	0,5	24,93	4,993	ZYN	M
4	1	40,01	7,619	ZYN	M
4	2	15,44	1,714	ZYN	M
4	4	<LLOQ[1]	-	ZYN	M
4	6	5,13	1,649	ZYN	M
4	10	<LLOQ[1]	-	ZYN	M
4	24	0	0	ZYN	M
4	0	0	0	ZYN Salt	M
4	0.25	6,16	0.04	ZYN Salt	M
4	0.5	88.52	1.078	ZYN Salt	M
4	1	194.61	11.198	ZYN Salt	M
4	2	231.65	9.903	ZYN Salt	M
4	4	115.85	9.482	ZYN Salt	M
4	6	53.71	5.693	ZYN Salt	M
4	10	6.54	1.827	ZYN Salt	M
4	24	0	0	ZYN Salt	M
5	0	0	0	ZYN	M
5	0.25	<LLOQ[1]	-	ZYN	M
5	0.5	23.49	0.77	ZYN	M
5	1	38.92	1.037	ZYN	M
5	2	35.18	1.36	ZYN	M
5	4	33.38	1.955	ZYN	M
5	6	11.92	2.882	ZYN	M
5	10	<LLOQ[1]	-	ZYN	M
5	24	<LLOQ[1]	-	ZYN	M
5	0	0	0	ZYN Salt	M
5	0.25	5.65	1.25	ZYN Salt	M
5	0.5	89.36	4.62	ZYN Salt	M
5	1	144.35	3.818	ZYN Salt	M
5	2	89.53	7.852	ZYN Salt	M
5	4	42.15	1.362	ZYN Salt	M

5	6	15.48	3.128	ZYN Salt	M
5	10	<LLOQ[1]	-	ZYN Salt	M
5	24	<LLOQ[1]	-	ZYN Salt	M
6	0	0	0	ZYN	F
6	0.25	<LLOQ[1]	-	ZYN	F
6	0.5	4.28	0.644	ZYN	F
6	1	23.85	2.573	ZYN	F
6	2	15.54	1.033	ZYN	F
6	4	29.1	1.591	ZYN	F
6	6	22.07	0.576	ZYN	F
6	10	<LLOQ[1]	-	ZYN	F
6	24	<LLOQ[1]	-	ZYN	F
6	0	0	0	ZYN Salt	F
6	0.25	0	0	ZYN Salt	F
6	0.5	50.62	2.58	ZYN Salt	F
6	1	75.33	6.216	ZYN Salt	F
6	2	77.45	6.893	ZYN Salt	F
6	4	26.38	1.846	ZYN Salt	F
6	6	12.56	2.4	ZYN Salt	F
6	10	<LLOQ[1]	-	ZYN Salt	F
6	24	0	0	ZYN Salt	F
7	0	0	0	ZYN	F
7	0.25	17.49	4.357	ZYN	F
7	0.5	43.67	4.082	ZYN	F
7	1	57	3.096	ZYN	F
7	2	50.93	1.608	ZYN	F
7	4	35.19	2.09	ZYN	F
7	6	22.39	0.954	ZYN	F
7	10	8.2	0.59	ZYN	F
7	24	0	0	ZYN	F
7	0	0	0	ZYN Salt	F
7	0.25	37.81	0.587	ZYN Salt	F
7	0.5	88.24	0.368	ZYN Salt	F
7	1	111.56	7.14	ZYN Salt	F
7	2	70.92	1.307	ZYN Salt	F
7	4	37.91	5.641	ZYN Salt	F
7	6	31.95	1.548	ZYN Salt	F
7	10	18.22	3.802	ZYN Salt	F
7	24	0	0	ZYN Salt	F
8	0	0	0	ZYN	F
8	0.25	0	0	ZYN	F
8	0.5	0	0	ZYN	F
8	1	0	0	ZYN	F
8	2	29	1.149	ZYN	F
8	4	10.51	0.483	ZYN	F
8	6	<LLOQ[1]	-	ZYN	F

8	10	<LLOQ[1]	-	ZYN	F
8	24	0	0	ZYN	F
8	0	0	0	ZYN Salt	F
8	0.25	10.68	1.57	ZYN Salt	F
8	0.5	17.84	0.688	ZYN Salt	F
8	1	70.14	3.073	ZYN Salt	F
8	2	78.33	1.313	ZYN Salt	F
8	4	38.05	2.061	ZYN Salt	F
8	6	<LLOQ[1]	-	ZYN Salt	F
8	10	<LLOQ[1]	-	ZYN Salt	F
8	24	0	0	ZYN Salt	F
9	0	0	0	ZYN	M
9	0.25	<LLOQ[1]	-	ZYN	M
9	0.5	25.39	2.624	ZYN	M
9	1	54.45	0.567	ZYN	M
9	2	41.9	1.715	ZYN	M
9	4	12.27	0.484	ZYN	M
9	6	<LLOQ[1]	-	ZYN	M
9	10	<LLOQ[1]	-	ZYN	M
9	24	0	0	ZYN	M
9	0	0	0	ZYN Salt	M
9	0.25	10.3	1.788	ZYN Salt	M
9	0.5	81.24	6.422	ZYN Salt	M
9	1	96.23	4.166	ZYN Salt	M
9	2	101.2	6.467	ZYN Salt	M
9	4	50.07	3.671	ZYN Salt	M
9	6	<LLOQ[1]	-	ZYN Salt	M
9	10	<LLOQ[1]	-	ZYN Salt	M
9	24	0	0	ZYN Salt	M
10	0	0	0	ZYN	F
10	0.25	15.4	0.649	ZYN	F
10	0.5	21.58	1.253	ZYN	F
10	1	34.17	0.8	ZYN	F
10	2	60.16	8.183	ZYN	F
10	4	49.67	0.845	ZYN	F
10	6	40.21	3.167	ZYN	F
10	10	21.41	0.267	ZYN	F
10	24	0	0	ZYN	F
10	0	0	0	ZYN Salt	F
10	0.25	22.25	2.001	ZYN Salt	F
10	0.5	56.87	1.008	ZYN Salt	F
10	1	76.22	1.655	ZYN Salt	F
10	2	93.75	4.798	ZYN Salt	F
10	4	69.77	2.125	ZYN Salt	F
10	6	39.28	0.424	ZYN Salt	F
10	10	25.74	1.146	ZYN Salt	F

10	24	<LLOQ[1]	-	ZYN Salt	F
11	0	0	0	ZYN	M
11	0.25	0	0	ZYN	M
11	0.5	8.01	2.177	ZYN	M
11	1	15.82	1.317	ZYN	M
11	2	29.04	1.247	ZYN	M
11	4	25.53	3.414	ZYN	M
11	6	9.01	0.474	ZYN	M
11	10	<LLOQ[1]	-	ZYN	M
11	24	0	0	ZYN	M
11	0	0	0	ZYN Salt	M
11	0.25	0	0	ZYN Salt	M
11	0.5	17.39	2.821	ZYN Salt	M
11	1	75.6	5.679	ZYN Salt	M
11	2	106.66	6.71	ZYN Salt	M
11	4	50.1	2.788	ZYN Salt	M
11	6	16.16	2.465	ZYN Salt	M
11	10	<LLOQ[1]	-	ZYN Salt	M
11	24	0	0	ZYN Salt	M
12	0	0	0	ZYN	M
12	0.25	0	0	ZYN	M
12	0.5	0	0	ZYN	M
12	1	11.72	0.69	ZYN	M
12	2	18.4	0.974	ZYN	M
12	4	9.98	0.312	ZYN	M
12	6	<LLOQ[1]	-	ZYN	M
12	10	<LLOQ[1]	-	ZYN	M
12	24	0	0	ZYN	M
12	0	0	0	ZYN Salt	M
12	0.25	4.95	1.025	ZYN Salt	M
12	0.5	34.27	1.884	ZYN Salt	M
12	1	47.59	1.462	ZYN Salt	M
12	2	30.61	1.028	ZYN Salt	M
12	4	5.45	1.007	ZYN Salt	M
12	6	<LLOQ[1]	-	ZYN Salt	M
12	10	0	0	ZYN Salt	M
12	24	0	0	ZYN Salt	M

Mangiferin was detected but below the lower limit of quantification (LLOQ; 5 ng/mL). [1]The M5 method of Beal et al. (2001) was adopted to fit the pharmacokinetic curves [47]. A value of 0 was assigned to those samples in which mangiferin was not detected (below the method limit of detection or; <LOD).
